# Adaptation and psychometric properties of the Norwegian version of the heart continuity of care questionnaire (HCCQ)

**DOI:** 10.1186/s12874-019-0706-z

**Published:** 2019-03-18

**Authors:** Irene Valaker, Bengt Fridlund, Tore Wentzel-Larsen, Heather Hadjistavropoulos, Jan Erik Nordrehaug, Svein Rotevatn, Maj-Britt Råholm, Tone M. Norekvål, On behalf of the CONCARD Investigators

**Affiliations:** 1 Faculty of Health and Social Sciences, Western Norway University of Applied Sciences, Førde, Norway; 2 Department of Heart Disease, Haukeland University Hospital, Bergen, Norway; 3 Centre of Interprofessional Collaboration within Emergency care (CICE), Linnaeus University, Växjö, Sweden; 4 Centre for Clinical Research, Haukeland University Hospital, Bergen, Norway; 5 Centre for Child and Adolescent Mental Health, Eastern and Southern, Oslo, Norway; 6 Norwegian Centre for Violence and Traumatic Stress Studies, Oslo, Norway; 7 Department of Psychology, University of Regina, Regina, Saskatchewan Canada; 8 Department of Clinical Science, Faculty of Medicine, University of Bergen, Bergen, Norway; 9 Department of Cardiology, Stavanger University Hospital, Stavanger, Norway; 10 Norwegian Registry for Invasive Cardiology, Bergen, Norway

**Keywords:** Cardiac care, Confirmatory factor analysis, Continuity of care, Cross-cultural adaptation, Patient perspective, Percutaneous coronary intervention, Psychometric properties, Validation

## Abstract

**Background:**

Continuity of cardiac care after hospital discharge is a priority, especially as healthcare systems become increasingly complex and fragmented. There are few available instruments to measure continuity of cardiac care, especially from the patient perspective. The aim of this study was (1) to translate and adapt the Heart Continuity of Care Questionnaire (HCCQ) to conditions in Norway, and (2) to determine its psychometric properties in self-report format administered to patients after percutaneous coronary intervention (PCI).

**Methods:**

The HCCQ was first translated into Norwegian from the original English version, following a widely used cross-cultural adaptation process. Data were collected before hospital discharge and in a follow-up after 2 months. To assess psychometric properties, a confirmatory factor analysis (CFA) was performed and three aspects of construct validity were evaluated: structural validity, hypotheses testing and cross-cultural validation. Internal consistency of the HCCQ subscales was calculated using Cronbach’s alpha, while intra-class correlation (ICC) was used to assess test-retest reliability. Additionally, socio-demographic and patient-reported data were collected to correlate with HCCQ scores.

**Results:**

Of those included at baseline, 436 (76%) completed the questionnaires after 2 months. CFA suggested that the fit of the HCCQ data to a 3-factor model was modest (RMSEA = 0.11, CFI = 0.90, TLI = 0.90). However, convergent validity was satisfactory, based on existing research. Internal consistency was good, as indicated by its Cronbach’s alphas: total continuity of care (0.95); informational (0.93), relational (0.87), and management (0.89) continuity. The ICC for the total HCCQ score was 0.80 (95% CI [0.71, 0.87] *p* < 0.001). As indicated by negative care experiences (rated as 1 or 2 on the five-point scale), patients seemed to have limited knowledge about medical treatment, lifestyle modification and follow-up after PCI. Participation in cardiac rehabilitation and longer consultations with the general practitioner after hospital discharge were positively correlated with better continuity of care.

**Conclusions:**

Implementation of the HCCQ will likely support healthcare providers and researchers in identifying problem areas of continuity of cardiac care and in evaluating interventions aimed at improving continuity of care.

## Background

Continuity of care among different healthcare providers and from one healthcare setting to another is a priority for patients, healthcare providers and policymakers. It is becoming a major concern as healthcare systems become increasingly complex and fragmented [1–4].

Percutaneous coronary intervention (PCI) is the most widely performed procedure to treat patients with coronary heart disease [5, 6]. The treatment does not end with the PCI procedure, as continuity is a critical component of secondary prevention and favorable outcomes [6]. In addition, as healthcare policy is increasingly focusing on minimizing length of hospital stay, a trend has emerged in which patients are discharged earlier from hospital, with the recovery process being followed up in the primary care [5]. Thus, in a field where *interventional cardiology* technology continues to develop rapidly, continuity of care is a major concern for PCI patients.

Several definitions have been proposed to describe the concept of continuity of care [3, 7–9]. In a literature review, Haggerty et al. [10] identified three types of continuity: informational, relational, and management continuity. *Informational continuity* refers to the use of information from previous events to provide adequate care to the patient. *Relational continuity* is described as the ongoing relationship between a patient and one or more healthcare providers. *Management continuity* is viewed as the provision of complementary healthcare services with shared management. Even though continuity of care is important and a priority within healthcare, there are few instruments available to assess patients’ experiences with multiple dimensions of continuity of care [3, 8, 11, 12]. Such a measure has the potential to guide quality improvement initiatives related to continuity of care [3, 7, 9].

Four promising instruments have been employed to measure patients’ experiences with continuity of care; one of these is the Heart Continuity of Care Questionnaire (HCCQ) [13]. The HCCQ was the first to measure multiple dimensions of continuity of care specifically among cardiac patients. It also corresponds well to the three aspects of continuity of care that Haggerty et al. identified [10]. HCCQ has been reported to be a comprehensive, valid and reliable instrument for measuring continuity of care from the patient perspective in patients with congestive heart failure, atrial fibrillation or patients hospitalized for acute coronary syndrome [12, 14, 15]. Despite this, further attention on validity and reliability is warranted [16] to determine whether the HCCQ can be administered in a self-report format [12].

The use of a single instrument in cardiac care can help to crystallize conceptualizations of continuity of care, and as healthcare research has increasingly become international in scope it can also facilitate comparisons among findings in different countries [17]. The cross-cultural adaptation of an instrument requires a specific methodology to reach equivalence between the original source and target languages [18, 19]. The aim of this study was (1) to translate and adapt the HCCQ to conditions in Norway, and (2) to determine its psychometric properties in self-report format administered to patients after PCI.

## Methods

### Design

This methodological sub-study was part of a larger prospective multicenter, register-based study, the CONCARD^PCI^. The study population for this paper included patients from one university hospital. Procedures were consistent with ethical guidelines of the World Medical Association, Helsinki declaration [20]. Patients gave written informed consent. Confidentiality and the right to withdraw from the study were assured. The methodological sub-study was approved by the Norwegian Regional Committee for Ethics in Medical Research (REK 2015/57).

### Participants

The study included 436 patients of 571 included at baseline from June 2017 to March 2018.

Inclusion criteria were patients undergoing PCI, hospitalized 2 months earlier, ≥ 18 years of age, living at home at the time of inclusion, and having answered at least one baseline HCCQ question. Exclusion criteria were unable to speak Norwegian or unable to fill out the questionnaire due to reduced capacities. Institutionalized patients or patients who might likely die in less than a year were excluded. Additionally, patients undergoing PCI without stent implantation and patients undergoing PCI related to transcatheter aortic valve implantation or MitraClip® were not included. Finally, patients who were later readmitted to hospital were also excluded.

### Procedures

Retrospective baseline self-reports of demographic information and certain clinical data were obtained after PCI, but before discharge from hospital. Two months after discharge, questionnaires were mailed by post to all patients included in the study. The two-month time interval was chosen to allow for sufficient follow-up care and so that patients could give an adequate evaluation of early post-discharge continuity of care. A pre-stamped envelope was included and non-responders were reminded once if they did not response within a certain period of time. Pilot testing was done with a version of the HCCQ with 55 PCI patients before using the final adapted instrument in the main cohort study. A random sub-group of 95 patients was approached for a HCCQ test-retest after 2 weeks [18].

### Cross-cultural adaptation of HCCQ

A cross-cultural adaptation was completed to reach equivalence between the original source and the target Norwegian version [18, 21]. The translation process was conducted systematically in six steps as described by De Vet et al. [18] and Beaton et al. [21].

In any research where different cultures are involved, systematic measurement bias may occur that affects the results of the study. Polit and Yang et al. [22] introduced five levels of cross-cultural equivalence; a) conceptual equivalence concerns the extent to which the construct of interest exists in another culture and whether the construct has similar meaning, b) content equivalence concerns the cultural relevance of individual items for the focal construct within the culture under consideration, c) semantic equivalence is the extent to which the meaning of an item is the same in the target culture after translation as in the original version, d) technical equivalence concerns the equivalence of assumptions about the methods of instrument administration, and e) measurement equivalence concerns the comparability of various measurement properties in the original and translated version of a scale.

A critical review of the equivalence was discussed with an expert group including a physician in community medicine, a professor of cardiac nursing, two cardiac nurse specialists, and a professional translator. These reviewers had experience with translation procedures and were knowledgeable about continuity of care and issues in cardiology. In addition, a patient representative identified in collaboration with the Norwegian Heart and Lung Foundation provided input to the planning of the study and was also involved in the translation process. When asking the patient representative to comment on the instrument in general, the experts employed a cognitive pretesting framework [18].

### Translation procedure

**Fig. 1:**
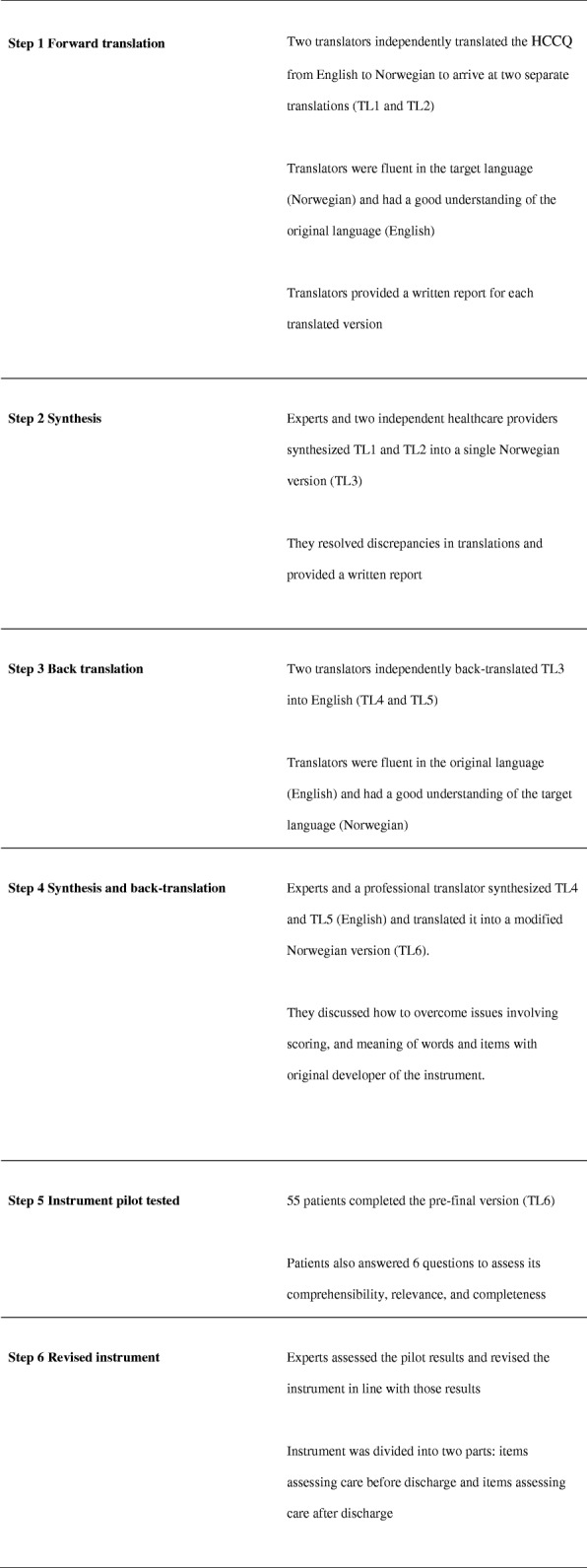
Steps for translation and cross-cultural adaptation of Heart Continuity of Care Questionnaire (HCCQ) into Norwegian

Figure 1 provides a summary of the overall translation procedure.

**Step 1.** Two forward translations of the English HCCQ were made. The experts selected two bilingual translators who had the target language (Norwegian) as their mother tongue. The translators worked independently, and both wrote a report that identified challenging phrases and described their rationale for final translation choices. Examples of difficult words or phrases to translate into Norwegian were “satisfied with the level of care,” “overall treatment plan,” “dietary needs,” and “open line of communication.” The two translations (TL1 and TL2) were compared and discrepancies were identified.

**Step 2.** The experts synthesized TL1 and TL2 into one consensus version (TL3) and described how they resolved discrepancies. Furthermore, the experts asked two healthcare providers familiar with the cardiac patient population to evaluate TL3 from a clinical perspective and to evaluate its face validity. Their recommendations were used to shape the final HCCQ. The experts discussed any differences in the translation and selected the best and most accurate version of the translated instrument.

**Step 3.** To further ensure the accuracy of the HCCQ, two persons who had a good understanding of English who also spoke Norwegian fluently independently translated TL3 back into English (TL4 and TL5). This back-translation process provided critical feedback on the vocabulary used in TL3 and achieved different aspects of cross-cultural validity.

**Step 4.** The experts and a professional translator synthesized TL4 and TL5, and agreed on a modified Norwegian version of the HCCQ (TL6). The experts also discussed semantic and technical equivalence with the original developer of the instrument from Canada: for example, timing of administration, scoring of the results, and meaning of certain words and items in the original version. The main challenge when translating the English HCCQ was to re-structure sentences from English to Norwegian to produce readily comprehensible questions. In order to increase the understanding of the items a few words were changed or small changes were made to the items of the questionnaire.

**Step 5.** A pre-final version (TL6) was sent to 100 patients, of which 55 answered. The responses were explored concerning how each person interpreted the items in the instrument and in particular identifying the proportion of missing items. After patients had completed the instrument, they were asked to answer six questions identifying words and phrases that might be difficult to understand and commented on the overall impression of the instrument.

**Step 6.** The experts evaluated the adapted HCCQ (TL6) and the patients’ experiences and answers to the six questions. In the pre-final version, 3–7% had missing data on at least one question. Five patients thought some questions were repeated and that the instrument was too extensive. None of the patients reported any difficulties understanding the response categories. Two patients found it challenging to answer items on whether healthcare providers communicated effectively after discharge. Three patients did not know which hospital to evaluate for the questions, since they had visited several. Therefore, the experts decided to separate the HCCQ into two sections: one section addressing care before discharge, and another section addressing care after discharge. Additionally, they decided to elaborate on the introduction to the HCCQ to clarify which situation(s) should be evaluated. After the translation and adaptation process, the instrument was evaluated for psychometric properties and measurement equivalence.

### Study instruments

#### The heart continuity of care questionnaire (HCCQ)

The original English HCCQ is a 33-item self-report questionnaire that assesses patients’ experiences with continuity of cardiac care. It measures this continuity along three dimensions: informational (17 items), relational (10 items), and management (6 items) [12, 15]. From the perspective of the patient, the self-report instrument covers major topics in cardiac care: heart condition explained, communication among healthcare providers, preparation for discharge, post-hospital care, post-hospital review of treatment, consistent information, information on medications, and knowledge on physical and dietary needs. Items were rated on a 5-point Likert-type scale from 1 (strongly disagree) to 5 (strongly agree), as well as the option to choose ‘not applicable’. Missing data was handled with half rule; using the mean of the answered items in the subscale, if at least half of that subscale had been answered [22]. The English version of the HCCQ is reported to be comprehensive, valid, and reliable. Cronbach’s alpha for the total instrument was 0.95 and the internal consistencies for subscales ranged from 0.80–0.93 [12, 15].

#### Nordic patient experiences questionnaire (NORPEQ)

NORPEQ is a brief eight-item tool that measures important aspects of patients’ experiences with healthcare interactions [23]. Here, the six items describing concern patients’ experiences with healthcare providers were used. These items assessed whether information provided by physicians was understandable, physicians’ and nurses’ professional skills and nursing care, whether the physicians and nurses were interested in the patients’ problems, and information related to diagnostic tests. Items are rated on a 5-point Likert-type scale from 1 (not at all) to 5 (to very large extent). The scores on these six items are summed to produce an overall subscale score ranging from 0 to 100, where 100 indicates the best possible experience of care [23]. Missing data were handled using the half-rule [22]. NORPEQ has good validity and reliability showing a Cronbach’s alpha of 0.85 and is recommended for cross-national comparisons of healthcare experiences in Nordic countries [24].

#### The patient experiences during hospitalisation in somatic hospitals

National surveys are carried out regularly concerning patients having received inpatient specialist health care at Norwegian hospitals. The goal is to obtain information on patients’ experiences when hospitalized in somatic hospitals [25]. In this study, a single item from this survey was used: “Do you feel that the hospital has cooperated well with the general practitioner about what you have been hospitalised for?” The item was rated on a 5-point Likert-type scale, ranging from 1 (not at all) to 5 (to a very large extent).

#### World Health Organization quality of life abbreviated (WHOQOL-BREF)

The WHOQOL-BREF is a 26-item scale that assesses a person’s perception of quality of life [26]. The World Health Organization (WHO) defines quality of life as follows: *“*…*individuals’ perception of their position in life in the context of the culture and value systems in which they live, and in relation to their goals, expectations, standards and concerns.” O*ne item that is a global measure of overall quality of life was used: “How would you rate your quality of life?” This item was rated on a 5-point Likert scale, ranging from 1 (very poor) to 5 (very good). WHOQOL-BREF has acceptable psychometric properties in the Norwegian population [26], and has previously been used to assess quality of life in patients with coronary heart disease [27].

#### RAND 12-item short form health survey (RAND-12)

RAND-12 is an abbreviated version of the RAND-36 [28]. The 12-item generic self-report instrument was developed to reproduce the physical and mental component summary scores of the RAND-36. The RAND-12 has three to five response levels, with higher scores reflecting better self-reported health. The RAND-12 is a valid and reliable instrument when used in the Norwegian population [29, 30].

#### Characteristics of the study population

Socio-demographic data (marital status, education, work status) were obtained by self-report, along with questions developed specifically for this study: duration of hospital stay, participation in cardiac rehabilitation (CR), first meeting with a general practitioner (GP) after PCI and how long the patient has been with their current GP.

### Expected relationships and subgroup means

The hypotheses were formulated in advance, i.e. before data collection based on an underlying theoretical model, expected differences between subgroups of patients and relationships with similar constructs. It was hypothesized that the sub-scales of the HCCQ instrument would reproduce the dimension structure defined by Haggerty et.al. [10]. Furthermore, it was hypothesized that patients’ CR participation are positively related to HCCQ scores based on Riley et al.’s findings [14]. They found that patients had better perceptions of continuity of care if they participated in CR. Although there is insufficient evidence linking patients’ experiences of continuity of care with other characteristics, it was expected that scores would differ according to the sociodemographics of the patients (e.g., age, educational level, gender and cohabitation status) [14, 31, 32]. It was expected that patients who had longer hospital stays would have lower HCCQ scores [33]. Conversely, it was expected that patients who consulted their GP soon after their hospital discharge would have higher HCCQ scores [34, 35]. As indication of construct validity, HCCQ was expected to have a positive moderate association with NORPEQ and the item measuring patients’ experiences with cooperation between the hospital and their GP. The correlation between the HCCQ and RAND-12 was predicted to be weak (r = 0.10–0.30), as these are thought to assess two different constructs [12].

### Statistical analyses

Descriptive statistics were used to summarize patients’ sociodemographic characteristics, clinical data, and HCCQ scores. Item means, standard deviations, missing rates and the percentage of “not applicable” for each item were calculated, although items were ordinal, to be able to compare with the original English version. Similarly to Hadjistavropoulos et al. [12] items that had mean score below 3.75 and at least 25% of the patients indicating negative care experiences (rated as 1 or 2 on the 5-point scale) were identified. Floor and ceiling effects were estimated. Non-parametric tests were used for ordinal variables and parametric tests for continuous variables. Continuous variables were characterised by means and standard deviations. Pearson correlations were used between continuous variables, while Spearman correlations were used when ordinals variables were involved. A strong correlation was operationally defined as r > 0.70, moderate to substantial as 0.30–0.70 and weak as < 0.30, in absolute value [18].

In general, three different types of validity can be distinguished: content validity, criterion validity and construct validity. Criterion validity involves comparing the newly developed measure to “a gold standard” [18, 22]. Currently there is no validated instrument for comprehensive assessment of continuity of care in Norway. In the absence of such a gold standard, a reasonable alternative is to compare the HCCQ with existing instruments having similar constructs. Three aspects of construct validity were evaluated for the HCCQ: hypotheses testing (convergent/discriminant validity), structural validity, and cross-cultural validation. Convergent validity was tested by correlating the HCCQ instrument with RAND-12 and NORPEQ, using Pearson correlation coefficients. The association between HCCQ and gender was evaluated by using an independent t-test, while between HCCQ and age by using Pearson correlation.

According to Polit and Yang (2016) five types of factorial invariance can be assessed; dimensional, configural, metric, scalar and strict factorial. Such tests require raw data from both groups being compared. However, data from the original English version was not available why dimensional and configural invariance were evaluated using data from the adapted/translated scale. Confirmatory factor analysis (CFA) was used for evaluating the three-factor structure of the original HCCQ instrument. To do this, the following fit indices were used: (a) the root mean squared error of approximation (RMSEA) (preferably < 0.06); (b) Tucker-Lewis index (TLI) (preferably > 0.95); and (c) comparative fit index (CFI) (preferably > 0.95) [18]. A weighted least squares estimator (WLMSV) was used. The WLMSV is a robust estimator that does not assume normally distributed variables and is appropriate for ordinal variables [36].

**Fig. 2:**
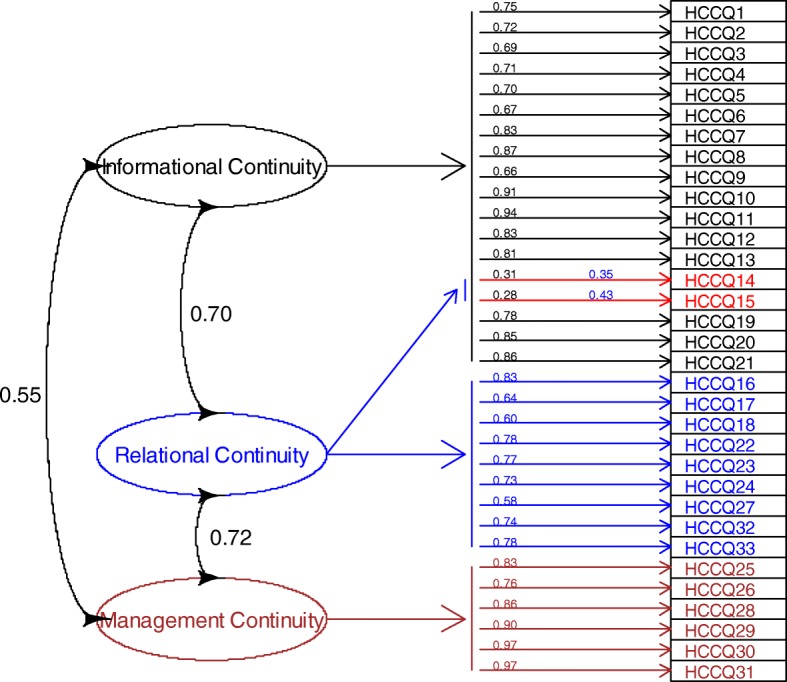
Hypothesized first-order CFA model

There were three hypothesized continuity of care factors, which were included as latent variables underlying the variation and covariation between the observed variables. This hypothesized model for these relationships is presented schematically in Fig. 2. Based on theory and empirical research, the factors are called informational continuity (factor 1), relational continuity (factor 2), and management continuity (factor 3).

Because explorative factor analysis performed by Hadjistavropoulos et al. [12] showed that item 14 and 15 of the original HCCQ had cross loadings on more than one factor, a second model was constructed to include these cross loadings. In the first model, items are related to one factor only, while in the alternative model two items (item 14 and 15) are both related to informational and relational continuity. The two models were estimated and compared with the results of Hadjistavropoulos et al. [12]. Furthermore, an additional analysis was carried out in which item 14 and 15 were removed from the model. In Fig. 2, the arrows from the factors to the items represent factor loadings. The bi-directional arrow between each of the three factors indicates our assumption that the three factors are intercorrelated.

Internal consistency of the HCCQ was evaluated using Cronbach’s alpha for total continuity of care and for each extracted domains; alpha values of > 0.70 were considered to reflect satisfactory internal consistency. Test-retest reliability was evaluated by using intraclass correlation (ICC) coefficients of 95 patients’ results obtained at a 2-week retest interval [18]. The ICC for agreement was used to get the absolute agreement between repeated measurements. For assessing measurement error, limits of agreement (Bland and Altman method) [37] and standard error of measurement were used [18]. Reliable change was estimated using the smallest detectable change (SDC).

Little et al. [38] recommend sample sizes that are a bit larger than 100 observations for single factor structural equation models (SEM). However, several factors influence the sample requirements of an SEM model, including the quality of the data to produce accurate estimates of the sufficient statistics, heterogeneity and representativeness of the sample, precision of the instrument, and model complexity [39]. A sample size of 436 was considered adequate for the CFA. A two-sided *p*-value of < 0.05 was considered to be statistically significant. SPSS (IBM Corp. Released 2016. IBM SPSS Statistics for Windows, Version 24.0. Armonk, NY: IBM Corp.) was used for calculating summary statistics and correlations, and for conducting statistical comparisons. Mplus (Computer software, 1998–2018, version 7) by B.O. Muthén, L.K. Muthén, was used to perform SEM.

## Results

### Characteristics of study population

Of the 571 patients included at baseline, 436 (76%) completed the questionnaires at the 2-month post-discharge assessment. These 436 patients ranged in age from 30 to 92 years, with a mean age of 66 years (Table 1). A total of 74% of patients were men, 82% lived with others, and 28% had completed a high school education. With regard to employment, 33% worked full time and 51% were retired. The majority of the patients (57%) were hospitalized for 3 days or less, and 3 out of 4 were discharged to home directly.

**Table 1:** Characteristics of PCI patients who completed baseline and follow-up assessments (*N* = 436)^a^

	N (%) or Mean (SD)
Gender	
Male	323 (74%)
Female	113 (26%)
Mean age in years (SD)	66.4 (10.3)
Cohabital status	
Living with others	335 (82%)
Living alone	74 (18%)
Education level attained	
Primary School	102 (24%)
Trade school	154 (37%)
High School	47 (11%)
College/University	118 (28%)
Employed	
Work full-time	131 (33%)
Retired	204 (51%)
Other (work part-time, sick leave, disability pension, seeking employment)	65 (16%)
Duration of hospital stay	
1 day	94 (23%)
2 days	75 (18%)
3 days	66 (16%)
4 days	65 (16%)
> 4 days	115 (28%)
Transferred	
Discharged to home	313 (75%)
Transferred to another hospital	89 (21%)
Other	16 (4%)
Cardiac rehabilitation	
Yes	172 (41%)
No	244 (59%)
First post-discharge meeting with GP	
Before 4 weeks	264 (64%)
Within 4–8 weeks	88 (21%)
Have not visited the GP	59 (14%)
Duration of patient’s relationship with current GP	
Below 1 year	61 (14%)
1–2 years	42 (10%)
Between 2 and 4 years	67 (16%)
More than 5 years	253 (60%)
Sufficient time in consultations with GP	
Not at all	12 (3%)
To a small degree	13 (3%)
To some degree	92 (22%)
To a large degree	215 (52%)
To a very large degree	85 (20%)

^a^Total counts (N) for a given variable may not necessarily sum to 436, because some patients failed to answer some items

### Item and sum score analysis of HCCQ

Descriptive statistics of the 33 items of the HCCQ are presented in Table 2. Several items on the HCCQ had mean of < 3.75 (i.e., rated 1 or 2), indicating negative care experiences. For the response categories “strongly disagree” and “somewhat disagree,” on several items of patients’ care experiences stand out as frequent. For instance, 56% of the patients stated that they were not adequately informed about how their heart condition would influence on their lifestyle, and 54% of the patients stated that they were not adequately informed about the types of physical activity they should engage in or avoid. Similarly, 54% of patients reported that their treatment had not been adequately reviewed by their physician following discharge. The mean subscale score on the HCCQ for informational continuity was 3.26, for relational continuity 3.69, and for management continuity 2.49. The total mean score was 3.24 (SD = 0.82) (Table 3). The HCCQ total floor effect was 0% and ceiling was 1.7%. On sub-scales, information continuity floor was 0% and ceiling 4%, relational continuity floor was 0.5% and ceiling 6.8%, and management continuity floor was 4% and ceiling 4.5%.

**Table 2:** Item analysis of the 33 items in the Heart Continuity of Care Questionnaire (HCCQ)

HCCQ item number and descriptions	N	Mean	SD	Strongly or somewhat disagree (%)	Not applicable	Missing (%)
1. Provided with information	425	4.03	1.13	12	1	10
2. Condition clearly explained	423	4.22	1.06	9	2	11
3. Told what symptoms to expect	403	3.14*	1.32	32	12	21
4. Given opportunity to ask questions	411	4.11	1.11	9	11	14
5. Medication explained.	412	4.05	1.22	13	12	12
6. Told when and how to take medication	408	4.48	0.99	6	11	17
7. Told about potential side effects	412	2.63*	1.33	48	8	16
8. Told what to do if side effects occurred	412	2.24*	1.25	61	9	15
9. Given same information about medications	399	3.40*	1.33	23	21	16
10. Told what changes to make to diet	403	2.48*	1.38	53	18	15
11. Instruction to plan own daily meals	403	2.34*	1.35	61	15	18
12. Explained influence on lifestyle	403	2.41*	1.33	56	16	17
13. Explained physical activity	409	2.51*	1.43	54	11	16
14. Providers communicated well in hospital	403	4.10	1.01	5	16	17
15. Providers communicated well in planning move	411	3.95	1.13	10	11	14
16. Providers communicated well after discharge	363	3.43*	1.16	15	47	26
17. Providers obtained needed information from other providers	382	3.91	1.01	5	27	27
18. Family physician involved in care	394	3.45*	1.39	24	20	22
19. Well prepared for discharge	418	3.41*	1.28	26	4	14
20. Told what symptoms to call doctor about	413	2.83*	1.44	45	5	18
21. Consistent information about symptoms to seek help for	385	2.93*	1.43	38	24	27
22. Knew who to contact about problems after discharge	404	3.18*	1.60	38	12	20
23. Satisfied with care after discharge	383	3.99	1.21	11	32	20
24. After discharge, could access services	362	3.63*	1.31	18	53	21
25. Reviewed treatment plan	378	2.60*	1.63	54	30	21
26. Regularly scheduled appointments	389	3.06*	1.70	41	28	28
27. Doctor is aware of blood test results	414	4.19	1.15	8	12	19
28. Reviewed heart medication	401	2.91*	1.74	47	19	10
29. Explained again how medication should be taken	399	2.56*	1.67	57	22	16
30. Explained again potential side effects	394	1.95*	1.31	73	24	15
31. Explained again what to do about side effects	394	1.85*	1.25	75	24	18
32. Consistent information from doctors	383	3.61*	1.34	17	32	18
33. Consistent information from doctors and other providers	378	3.55*	1.30	18	37	21

^a^Scores range from 1 to 5, with higher scores denoting more positive continuity experiences

^*^Item represent an area of concern (i.e., mean < 3.75). Patients had the option to choose “not applicable” (e.g., did not receive services following discharge)

**Table 3:** Mean scores for the continuity of care domains of the Heart Continuity of Care Questionnaire (HCCQ)

Domain	Mean	SD	N
Information continuity	3.26	0.89	420
Relational continuity	3.69	0.85	410
Management continuity	2.49	1.26	402
HCCQ total	3.24	0.82	419

### Psychometric analyses

#### Structural validity

Table 4 presents results of the CFA of the HCCQ. The result of the first CFA was as follows: Chi-square (χ^2^)/ Degree of freedom (df) =3047/492; RMSEA = 0.11; CFI =0.90 and TLI = 0.89. Standardized factor loadings ranged from 0.57 to 0.97 (*p* < 0.001). In the alternative model (Table 4) with items 14 and 15 related to both informational and relational continuity, Chi-square (χ^2^)/ Degree of freedom (df) was somewhat reduced at 2969/490, but the fit indices did not appreciably improve; the RMSEA = 0.11; CFI = 0.90 and TLI = 0.90. Apart from items, 14 and 15, standardized factor loadings ranged from 0.60 to 0.97; for items 14 and 15, the loadings ranged from 0.28 to 0.43 (all p < 0.001). The fit indices reflected the fact that the structure was not well represented by the hypothesized 3-factor model [40]. The additional analysis, in which item 14 and 15 were removed, did not improve the fit appreciably: Chi-square (χ^2^)/ Degree of freedom (df) =2712/431; RMSEA = 0.11; CFI =0.91 and TLI = 0.90. Standardized factor loadings ranged from 0.58 to 0.97 (p < 0.001).

**Table 4:** Results of Confirmatory Factor Analysis of the Heart Continuity of Care Questionnaire (HCCQ)

	Factor Loading Matrices			
Item HCCQ	Information Continuity	Relational Continuity	Management Continuity	Tests of Model Fit
1	0.75			Chi-Square Test of Model Fit Value = 2969.15 Degree of freedom = 490 *p*-value < 0.001 Root Mean Square Error of Approximation Estimate = 0.11 Confidence interval = 0.10–0.11 Comparative fit index = 0.90 Tucker-Lewis index = 0.90
2	0.72			
3	0.69			
4	0.71			
5	0.70			
6	0.67			
7	0.83			
8	0.87			
9	0.66			
10	0.91			
11	0.94			
12	0.83			
13	0.81			
14	0.31^a^	0.35^a^		
15	0.28^a^	0.43^a^		
16		0.83		
17		0.64		
18		0.60		
19	0.78			
20	0.85			
21	0.86			
22		0.78		
23		0.77		
24		0.73		
25			0.83	
26			0.76	
27		0.58		
28			0.86	
29			0.90	
30			0.97	
31			0.97	
32		0.74		
33		0.78		

^a^Standardization model. Items 14 and 15 of the presented model load on both informational and relational continuity. These items had cross-loadings on more than one factor in the explorative factor analysis, according to the developer of the original English HCCQ [12]

#### Convergent and discriminant validity

Table 5 presents group statistics and correlations between HCCQ domain scores and values on patient-reported variables. It was hypothesized that domain scores might have systematic relationships with certain sociodemographic variables, such as age, gender, education level attained, and cohabital status, etc. [31, 32]. First, a significant difference between genders was found, with men scoring higher on items related to informational and relational continuity. Patients living alone scored lower on informational continuity, although not significant. It was also hypothesized that patients’ CR participation would have a positive impact on HCCQ scores [14]; the analyses revealed that patients who engaged in CR reported more positive experiences in terms of relational and management continuity compared to those who did not participate in CR.

**Table 5:** Group statistics and correlations between Heart Continuity of Care Questionnaire (HCCQ) domains, other instruments, and patients’ characteristics

	Informational Continuity	Relational Continuity	Management Continuity
Mean difference (*p*-value)			
Gender (Male = 0, Female = 1)	0.39 (< 0.001)	0.23 (0.022)	0.13 (0.359)
Live alone (No = 0, Yes = 1)	0.24 (0.070)	0.17 (0.172)	0.04 (0.835)
Participate in CR (No = 0)	−0.15 (0.103)	− 0.27 (0.002)	− 0.46 (< 0.001)
Correlations between HCCQ domains and patients’ variables (*p*-value)			
Age	−0.04 (0.446)	− 0.02 (0.757)	− 0.06 (0.247)
Education level attained	0.02 (0.689)	−0,01 (0.902)	− 0.07 (0.192)
Duration of hospital stay	0.04 (0.450)	0.04 (0.463)	0.16 (0.001)
Time elapsed between discharge and first appointment with GP	−0.001 (0.978)	−0.13 (0.009)	− 0.19 (< 0.001)
Duration of relationship with current GP	0.03 (0.579)	0.02 (0.743)	0.02 (0.632)
Sufficient time in consultations with GP	0.19 (< 0.001)	0.39 (< 0.001)	0.26 (< 0.001)
Hospital cooperated with the GP	0.40 (< 0.001)	0.61 (< 0.001)	0.50 (< 0.001)
NORPEQ	0.42 (< 0.001)	0.40 (< 0.001)	0.16 (0.001)
WHOQL-BREF	0.22 (< 0.001)	0.20 (< 0.001)	0.07 (0.161)
RAND-12			
mental component	0.20 (< 0.001)	0.20 (< 0.001)	0.12 (0.027)
physical component	0.13 (0.018)	0.14 (0.009)	0.09 (0.084)

Note: Hypotheses about possible relationships between patient characteristics and domain scores on the HCCQ

Abbreviations: *GP* general practitioner, *CR* cardiac rehabilitation, *NORPEQ* the Nordic Patient Experiences Questionnaire, *WHOQL* World Health Organization Quality of Life; RAND-12, Health Status Inventory; physical and mental component

There was also a weak positive correlation between management continuity and duration of hospital stay (r = 0.16). Patients who met with their GP soon after discharge reported better relational continuity (r = 0.13) and management continuity (r = 0.19). There were also weak-to-moderate positive correlations (r = 0.19 to r = 0.39) between the HCCQ and whether the patients felt they spent enough time in consultation with their GP. Another finding indicated that there were moderate positive correlations between continuity of care and the item identifying cooperation between the hospital and GP (r = 0.40 to r = 0.61). A weak-to-moderate positive correlation between the HCCQ and the score derived from the six items from the NORPEQ (r = 0.16 to r = 0.42) was found. The analyses also revealed a weak positive correlation between informational and relational continuity and quality of life (WHOQL-BREF). Furthermore, weak correlations were found between self-reported health (RAND-12) and informational and relational continuity. There were no significant associations between continuity of care and age, education, and length of treatment with their current GP (*p* ≥ 0.192).

#### Reliability

**Fig. 3:**
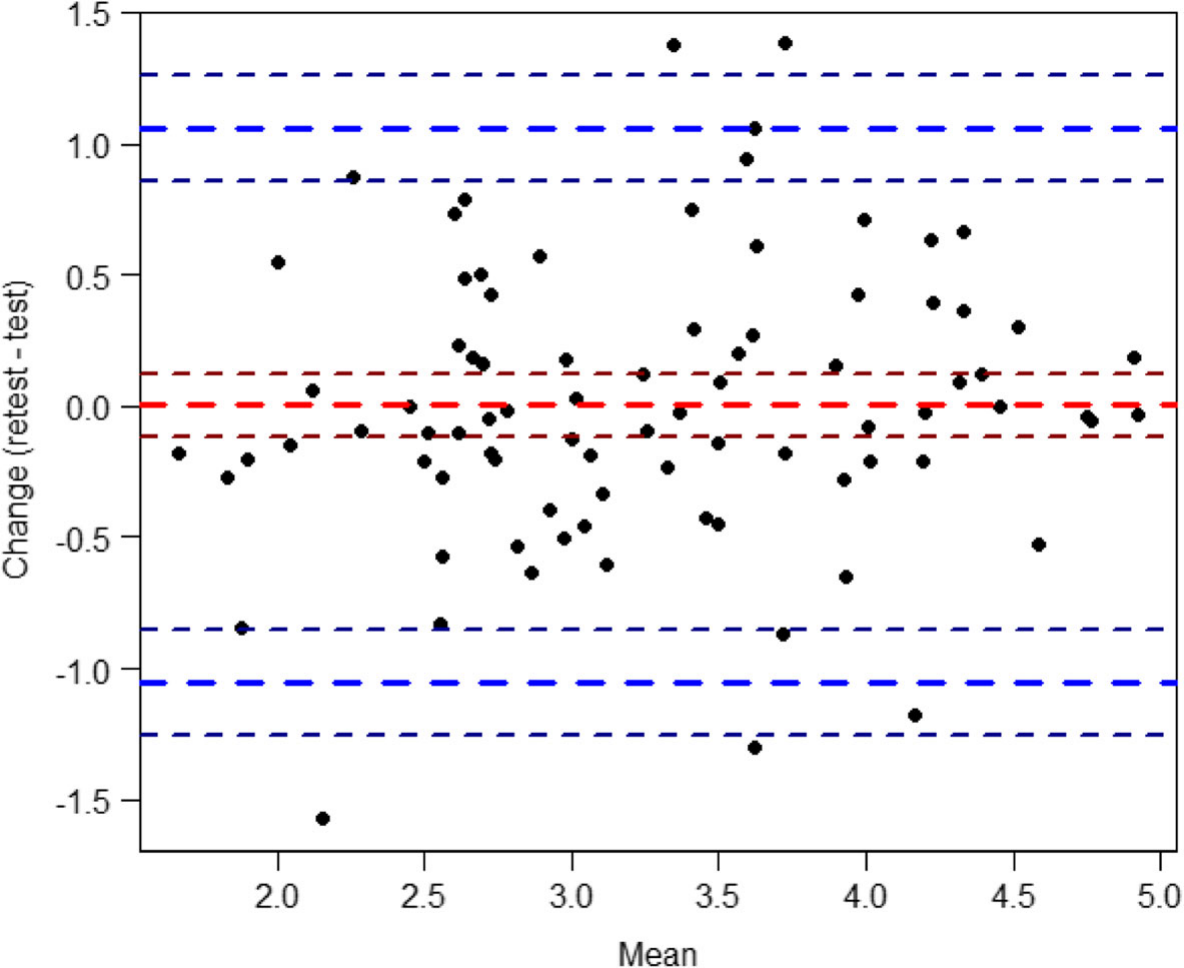
Bland-Altman plot for assessing measurement error. Text explaining Fig. 3 (legends): Each patient’s mean scores are plotted on the x-axis and the difference between scores on the y-axis. Blue dashed lines indicate the limits of the reference interval; thin dashed lines indicate 95% confidence intervals for the mean; and lines with shorter segments represent the reference limits.

Cronbach’s alpha for the three domains of informational, relational, and management continuity were 0.93, 0.87, 0,89, respectively; for the total scale Cronbach’s alpha was 0.95. The corresponding Cronbach’s alpha values at the 2-week retest were 0.94, 0.92, 0.92, and 0.96. The mean inter-item correlations within the HCCQ scale scores were 0.44, 0.40, and 0.59; at retest the correlations were 0.50, 0.54, and 0.66. The item-scale correlation was between 0.45–0.70. The ICC for informational continuity was 0.72 (95% CI [0.60, 0.81] *p* < 0.001); for relational continuity 0.84 (95% CI [0.77, 0.90] p < 0.001); for management continuity 0.82 (95% CI [0.73, 0.88] p < 0.001); and for the total score it was 0.80 (95% CI [0.71, 0. 87] p < 0.001). The mean systematic difference (dashed line in Fig. 3) was - 0.027. The limit of agreement was - 1.025 to 1.025. This difference was not statistically significant (*p* = 0.964). Standard error of measurement was 0.28 and SDC was ±1.05.

## Discussion

The psychometric properties of the Norwegian version of the HCCQ were evaluated for patients after PCI. The instrument had face and content validity by including relevant topics for cardiac care, and associations between similar and dissimilar scales showed satisfactory figures. However, CFA showed that the hypothesized model was not entirely adequate.

In order to meet the challenge of continuity of care, the healthcare services should involve patients to a greater extent when developing and evaluating the future healthcare [9, 32]. While patient integration contributes to achieving continuity of care, few tools to date have been developed and comprehensively validated to measure continuity of care from the cardiac patients’ perspective [3, 13]. This is especially the case for evaluating continuity of care in Norwegian healthcare systems.

In this study, the aim was to translate and adapt one such tool, the HCCQ [11, 12] and to determine its psychometric properties. The instrument will be helpful in providing new insights into the analysis of continuity of care across and within various healthcare levels. The results complement previous evaluations of the psychometric properties of the HCCQ, in this case for PCI patients interacting with the Norwegian healthcare system. The translation process included forward and backward translations and a pilot test, which produced an accurate translation and readily comprehensible questions that facilitated understanding of the Norwegian context. This comprehensive adaptation and pre-testing effort built a stable foundation for a valid psychometric evaluation. A test-retest ICC evaluation of the HCCQ confirmed that the measures were stable over time. CFA was used to evaluate the 3-factor structure of the HCCQ based on previous analysis [12]. This study showed that the translated and adapted version of the HCCQ would have good internal consistency in terms of informational, relational, and management continuity of care.

The literature search revealed that the HCCQ is suitable for measuring continuity of care in PCI patients. The expert group convened for the present study evaluated the content validity [22] of the HCCQ and determined it would be adequate for measuring the construct. Face validity [18] and other feasibility factors (e.g., ease of administration, usefulness) of the HCCQ were determined to be adequate by PCI patients and healthcare providers familiar with cardiac patients. Thus, it was deemed appropriate to translate and adapt the HCCQ for PCI patients speaking Norwegian. Cross-cultural instrument translation is a complex task that cannot be undertaken lightly without the risk of producing poor-quality adaptation [21]. The cross-cultural validation started with a translation process systematically following international guidelines [18, 21].

Importantly, not all words and phrases in the original English version of the HCCQ were easy to translate into comparable Norwegian words and phrases. Thus, the focus was to translate items that reflected the same concepts and were meaningful, clear, and relevant for the Norwegian context. Furthermore, it is important to translate the administrative and role-based hierarchies found in organizations [41]. For example, interdisciplinary primary health care teams have been established in provinces and territories in Canada. Additionally, the pharmacists play a more active role as part of the primary care team to ensure proper adherence to medications in the Canadian than in the Norwegian healthcare system [42] Therefore, some words in the instrument might be more relevant in the Canadian population. Squires et al. [41] suggest a more systematic approach for standardizing language translation processes including content validity indexing techniques.

The instrument was pre-tested with PCI patients, and these patients reported that the instrument was comprehensive and acceptable. However, a few patients thought the instrument was too extensive, suggesting that a shorter version may be more useful. In addition, a few patients in the pilot study reported that it was difficult to answer the item on whether healthcare providers communicated effectively after hospital discharge. With the final version of the HCCQ, 47 patients (11%) answered “not applicable” to this effective communication item, and 26 (6%) failed to answer the question. The literature suggests that patient perceptions related to continuity of care are strongly tied to how healthcare providers communicate with one another, both within and across sectors [43]. However, continuity in the delivery of care might not be visible to patients until they experience gaps in the quality of care given [44]. Furthermore, 14% of the patients reported that they had not visited their GP after discharge and were not able to judge these aspects of care. In this study patients were followed up 2 months after discharge in order to get solid experiences of the topic continuity of care and further on to avoid recalling bias. Nevertheless, the optimal time frame for evaluation of post-discharge continuity of care is currently unknown and requires further research.

Structural validity in psychometrics is defined as the degree to which patients’ scores on an instrument are an adequate reflection of the dimensionality of the construct being measured [18]. This can be assessed by factor analysis and CFA is preferred if a priori hypotheses about dimensions of the construct are available based on theory or previous analysis. With regard to the construct of continuity of care, Haggerty et al. [10] distinguished three dimensions, namely relational, informational, and management continuity. The HCCQ subscales correspond well to this three-factor model [12]. After the specified model for the HCCQ was identified, the model fit was determined. Among the resulting fit indices, the RMSEA was 0.11 (CI = 0.10–0.11), indicating a somewhat poor fit [45]. Two other indices used were CFI and TLI, which both measure the improvement in model fit when comparing the hypothesized model with a less restricted baseline model. Usually, a value greater than 0.90 is considered to be adequate, and 0.95 or greater is considered good. For this analysis of the HCCQ the CFI was 0.90 and the TLI was 0.90; values close to 1.0 indicate a well-fitting model. If a group of indexes provide contradictory indications about model fit, it is usually necessary to carefully re-evaluate the model. In this case, no empirically proposed modifications were regarded as reasonable. That being said, however, most disciplines recognize three types of continuity of care, as one interdisciplinary review of concepts and measures of continuity of care highlighted [10, 46].

An exploratory study of PCI patients showed that the HCCQ might not cover the overall set of items relevant to explaining continuity of care [35]. For example, some patients expressed that healthcare providers showed different levels of concern and interest about their cardiac disease, and they seemed to believe that ongoing relationships based on trust and confidence were important [35]. When the HCCQ was adapted to a generic population, items were added to better address relational continuity, including patients’ perceptions of satisfaction with emotional support, opportunity to discuss and ask questions, confidence in healthcare providers, sense of being understood, and feeling known by healthcare providers [33]. Continuity of care also includes patients’ healthcare experiences over time being perceived as being coherent and linked [10]. The HCCQ focuses on how patients experience the collaboration between specialist healthcare and GPs in primary healthcare [12]. Thus, issues important to the service delivery structure that directly relate to the patient, such as rehabilitation services and other supportive services, are less well addressed by items in the original HCCQ. These issues might also be of relevance in the adaptation and translation of the HCCQ into a Norwegian context and thereby affecting the structural validity.

The basic principle of construct validity is that hypotheses are formulated about associations of scores on the HCCQ with scores on other instruments measuring similar or dissimilar constructs, or differences in the instrument scores between subgroups of patients [18]. In this study, men scored better than women on relational and informational continuity (Table 5). There were no appreciable correlations between the HCCQ and age and education [40]. This finding is inconsistent with an earlier study suggesting that patients with higher education have higher expectations and judge quality of care more critically [47]. The study found a positive association between duration of hospital stay and management continuity of care. The shortened length of stay for patients after PCI, might limit the opportunities to achieve timeliness and complementarity of services [48].

With regard to hypothesis testing, patients who engaged in CR had more positive scores on management and relational continuity, similar to that in other studies [14]. This highlights that CR provides management continuity by timely, complementary services [4]. The period between hospital discharge and start of CR is very stressful for patients, and consequently CR organized by trained healthcare providers might support the experience of relational continuity [49]. In line with a previous study, the time elapsed between patient discharge and first access to a GP is associated with relational continuity [43]. Another significant finding was that having enough consult time with their GP after hospital discharge positively correlated with informational, relational, and management continuity. GPs are key collaborating partners in the healthcare system, and their ability and willingness to collaborate with patients are affected by organizational conditions [1]. Studies show that patients believe good communication with their physician requires sufficient time and quality of consultations [2, 50, 51]. However, the study did not find a substantial correlation between continuity of care and length of relationship with GPs. A previous study show that not all patients receiving care from a single GP thought they had a good personal relationship with the physician [43].

As expected, significant correlations between HCCQ and the six items in NORPEQ and the item regarding cooperation between hospital and GP were found. This is not surprising, since these two instruments have similar constructs as the HCCQ with regard to patients’ experiences with healthcare providers in hospital [24]. The study also confirmed previous findings that the HCCQ and RAND-12 are weakly correlated [12]. Generic instruments, such as the NORPEQ, are particularly useful for comparing outcomes for a wide range of patient groups. On the other hand, there is valid concern that they do less well at capturing areas of importance to specific patient populations [52]. The HCCQ is a disease-specific instrument and more clinically relevant for cardiac patients [12]. Moreover, questioning patients directly on actual experiences they had with the healthcare they received seems to describe the quality of care better than asking patients about satisfaction with the care [47].

The internal consistency of the three domains of the HCCQ, as assessed with Cronbach’s alphas, were comparable with that reported by Riley et al. [14] and that reported by Hadjistavropoulos et al. [12]. These similarities confirm that HCCQ has satisfactory internal consistency and the instrument is stable over time [18]. The Bland-Altman diagram showed a mean difference line close to zero and one lower and upper limit that are close to the mean difference, indicating substantial precision (less measurement error). Additionally, there were no major outliers and similar differences above and below the mean difference [22]. The standard error of measurement was also calculated and is not as affected as reliability coefficients by the sample within which the estimate is computed [22].

Individual item analysis of the HCCQ was particularly illuminating for identifying areas of concern to patients that would benefit from institutional review and quality improvement initiatives (cf. Table 2). The study highlights that achieving continuity of care for patients after PCI is challenging. For example, patients are not necessarily receiving adequate information about their medical treatment, possibilities for positive lifestyle modifications, and status of their physical condition. Additionally, patients were not being given consistent information about symptoms and when to contact healthcare providers.

Finally, the importance of patient participation in their healthcare, including sharing information, is highlighted in the most recent ESC/EACTS guidelines on Myocardial Revascularization [6]. Previous research indicates that healthcare providers at hospitals might ignore the critical component of post-hospital discharge care and the transition to home planning process [5, 53]. A global trend is for patients to discharge earlier after procedures, meaning there is reduced time for in-hospital education [5]. Moreover, patients are less receptive to learning. Inadequate education and poor discharge planning also seem to decrease patients’ adherence to treatment and prescribed lifestyle changes [1, 5].

Overall, the present study provides preliminary evidence that the HCCQ can highlight deficiencies in patients’ experiences of continuity of care across and within care levels, valuable information to help identify areas for healthcare improvement.

### Limitations of the study

The analysis of the translated and adapted HCCQ was specific to patients who recently underwent PCI and need to be tested for feasibility in self-report format for patients with other chronic cardiac conditions. It may also be beneficial to study the HCCQ using other modes of administration. In this study, the instrument was sent by post, but it is also possible that an equivalent form could be developed for online use, smart phones, or e-mail. CFA was used to determine the measurement equivalence of adapted and original measures. However, other relevant analyses could have been performed if there had been access to comparative data from the original instrument. Moreover, this study mainly focused on cross-cultural approaches for translating instruments and more research is needed to compare the HCCQ across healthcare systems and organizations. Additionally, future research should identify a cutoff score for each of the subscales that would indicate problems in continuity of care. The optimal post-discharge period for evaluating continuity of care can also be assessed systematically in future research. Recall bias at longer periods might distort or limit the amount of information that can be gained from patients. The HCCQ is only appropriate for patients who have no, or only minor, difficulties with communication. Follow-up of the patients is very important in a cohort study, and losses are an important source of bias in these types of designs. However, the CONCARD team thought carefully about the study population, and planning was given priority to avoid errors in sampling.

## Conclusions

The present study provides additional information on the psychometric properties of the HCCQ for patients after PCI. Cross-cultural validation was performed according to international guidelines. The internal consistency of the HCCQ was high, and ICC showed good agreement. However, the RMSEA suggests that the fit of data to the hypothesized model is not entirely adequate for fully capturing the theoretical components of informational, relational, and management continuity. Nevertheless, hypotheses about the constructs showed satisfactory results based on existing knowledge. For example, the results point to a positive relationship between continuity and CR participation and sufficient consultation time with the GP after discharge from hospital. Although more research is needed on the psychometric properties of the HCCQ, its use in this study identified problem areas in continuity of care, a critical step towards understanding and improving the quality of care.

## Abbreviations

CFA: Confirmatory factor analysis
CFI: Comparative fit index
CR: Cardiac rehabilitation
GP: General practitioner
HCCQ: Heart Continuity of Care Questionnaire
ICC: Intra-class correlation
PCI: Percutaneous coronary intervention
RMSEA: Root mean squared error of approximation
SEM: Structural equation modeling
TLI: Tucker-Lewis index
WLMSV: Weighted least squares estimator
